# Metachronous cutaneous squamous cell carcinoma in a young patient as the only presenting symptom to uncover Lynch syndrome with MLH1 Germline mutation

**DOI:** 10.1186/s13053-020-00155-w

**Published:** 2020-11-16

**Authors:** Karam Khaddour, Ryan C. Fields, Michael Ansstas, Ilana S. Rosman, George Ansstas

**Affiliations:** 1 Department of Medicine, Division of Medical Oncology, Washington University School of Medicine in Saint Louis, St. Louis, MO USA; 2 Section of Surgical Oncology, Department of Surgery, Washington University School of Medicine in Saint Louis, St. Louis, MO USA; 3 Alvin J. Siteman Cancer Center, St. Louis, MO USA; 4 Cedar Sinai Health System, Division of Gastroenterology, Los Angeles, California USA; 5 Division of Dermatology, Washington University School of Medicine in Saint Louis, St. Louis, MO USA; 6 Department of Pathology and Immunology, Washington University School of Medicine in Saint Louis, St. Louis, MO USA

**Keywords:** Lynch syndrome, Cutaneous squamous cell carcinoma, Germline mutation, MLH1, Pembrolizumab, Microsatellite instability

## Abstract

**Background:**

Cutaneous malignancies are rare complications of Lynch syndrome and can include Muir-Torre and Turcot syndromes that are associated with sebaceous gland tumors and keratoacanthomas. The incidence and clinical course of cutaneous squamous cell carcinoma have not been well documented in Lynch syndrome due to its rarity.

**Case presentation:**

A 49-year male presented with an enlarging groin skin lesion that was biopsed and demonstrated cutaneous squamous cell carcinoma for which he underwent a surgical resection. The patient experienced later a recurrence of cutaneous squamous cell carcinoma around the resected margins. Next generation sequencing of tumor tissue revealed mutations in MSH6 and MLH1, in addition to high microsatellite instability. The patient underwent pembrolizumab treatment with complete resolution of the cutaneous lesion in the groin, but subsequently developed a new mass in the right antecubital fossa shortly after discontinuation of pembrolizumab. Repeat biopsy of the antecubital fossa lesion revealed a recurrence of cutaneous squamous cell carcinoma. Germline mutation testing revealed MLH1 mutation, compatible with Lynch syndrome, and the patient restarted pembrolizumab which was associated with a complete response. The patient was referred for genetic counseling and cancer screening.

**Conclusions:**

Cutaneous squamous cell carcinoma, although rare, can be the initial presenting symptom in patients with Lynch syndrome. This association has been described in patients with germline mutations in MLH1. Lynch syndrome should be considered when evaluating young patients presenting with recurrent cutaneous squamous cell carcinoma with positive family history of malignancy and/ or without any identifiable risk factors for skin cancers, including those with a durable and rapid response to immunotherapy.

## Background

Lynch syndrome (LS) is an autosomal dominant disorder that was first described by Warthin in a family in Michigan state with a high susceptibility to develop cancers. This was further described in detail by Lynch in 1971. The etiology of LS involves a germline mutation in one of the DNA mismatch repair genes (*MLH1, MSH2, MSH6, PMS2*) or the *EPCAM* gene [1]. The clinical suspicion of LS is based on pre-defined criteria (Amsterdam Criteria) which focuses on family history of cancers in different generations and at an early age. Patients with LS are at an increased risk for developing a variety of cancers including cancers of the colon, uterus, small bowel, stomach, kidney, ureter, biliary tract, ovaries, brain and pancreas [2].

Skin tumors are uncommon manifestations of LS and have been described in the form of Muir–Torre syndrome which is associated with a higher risk of  sebaceous adenoma, sebaceous carcinoma or keratoacanthoma [3]. Cutaneous squamous cell carcinomas (cSCC) are rare in patients with LS, and there is paucity of literature describing their incidence and clinical course in this patient population. Here, we report a young patient who presented with advanced cSCC which was complicated by multiple recurrences in different locations, and was later diagnosed with LS due to MLH1 germline mutation.

## Case presentation

A 49-year-old male without significant past medical history presented after noticing a small raised papule in the left groin in 2016 that he initially thought to be a spider bite. He stated that over the subsequent year, the lesion grew in size and became more red and foul smelling. He was seen in multiple walk-in clinics and completed multiple courses of antibiotics. These interventions did not improve the lesion as it did continue to become more erythematous, painful, and ulcerated. This lesion was evaluated by plastic surgery service with subsequent biopsy in 2017 which demonstrated well differentiated squamous cell carcinoma (SCC). A staging computed tomography (CT) was performed which showed a 7.5 × 1.8 cm soft tissue mass on the left lower abdominal wall with ulceration and overlying skin thickening without areas of regionally advanced or metastatic disease. On further evaluation, the patient denied any prolonged sun exposure, radiation exposure, or history of immunosuppression. HIV and immune competence tests were normal. As part of his medical history, questioning revealed a family history of ovarian cancer in his mother (diagnosed in her late 50s) and colon cancer in his maternal uncle (unknown age at diagnosis).

The patient underwent a radical resection of the mass, resulting in a 15 cm diameter resection, and bilateral inguinal sentinel lymph node biopsies (SLNB). This demonstrated a 9 cm squamous cell carcinoma, moderately to poorly differentiated, and invasive to a depth of 16 mm with perineural invasion. The surgical margins were negative without nodal disease on SLNB. His wound was then closed with a skin graft.

**Fig. 1:**
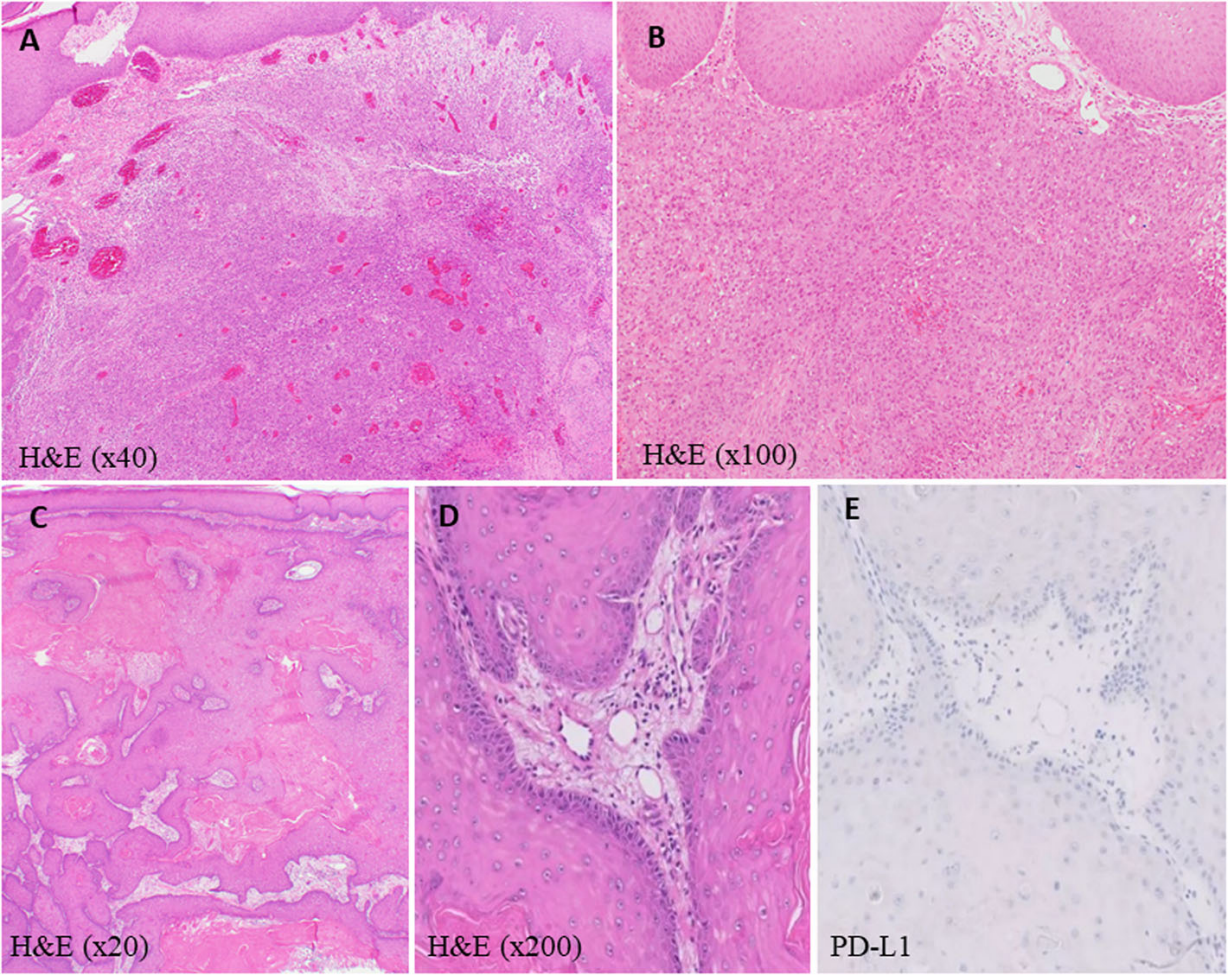
Histopathology of the cutaneous lesion showing **a** H&E × 40 of the groin lesion demonstrating proliferation of atypical keratinocytes in the dermis **b** groin lesions showing atypical keratinocytes containing abundance of cytoplasm with presence of keratin pearls (H&E × 100) **c** proliferation of atypical keratinocytes from a lesion in the antecubital fossa (H&E × 20) **d** High magnification of the arm lesion with cutaneous squamous cell carcinoma (H&E × 200) **e** skin lesion from the arm with PD-L1 expression < 1% of membranous tumor cell staining on immunohistochemical staining (DAKO PD-L1 28–8 clone)

**Fig. 2:**
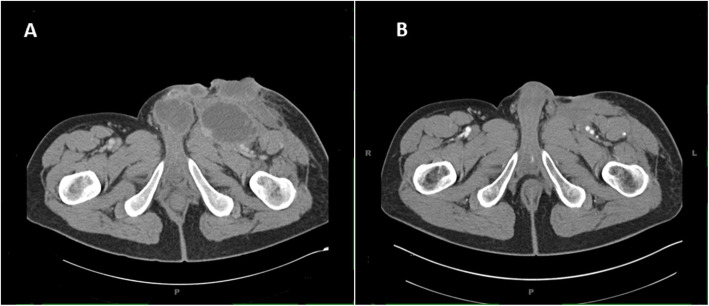
Computed tomography pelvis showing **a** peripherally enhancing low-attenuation lesions in the left lower pelvis extending into the proximal thigh with invasion of the adductor muscles and abutment of the penile shaft with possible invasion, there is also a subcutaneous lesion with ulceration and central necrosis. **b** Interval resolution of the previous low attenuating masses in the left inguinal region and the base of the penile shaft after initiation of pembrolizumab

After 7 months, the patient noted a new growth around his prior skin graft. Biopsy demonstrated recurrent cutaneous squamous cell carcinoma (Fig. 1). Tumor analysis with next generation sequencing (NGS) showed high tumor mutational burden (TMB) of 46 mutations per mega base and high microsatellite instability (MSI-H). There were multiple mutations including MSH6 (F1088fs*5) and MLH1 (loss of exon 4–8). Programmed death-ligand 1 (PD-L1) analysis by immunohistochemistry demonstrated PD-L1 expression of 30% on the tumor and 10% on the tumor-infiltrating immune cells. CT scan showed bulky disease in left lower pelvis extending to the thigh with invasion to the adductor muscles and abutment of penile shaft (Fig. 2).

**Fig. 3:**
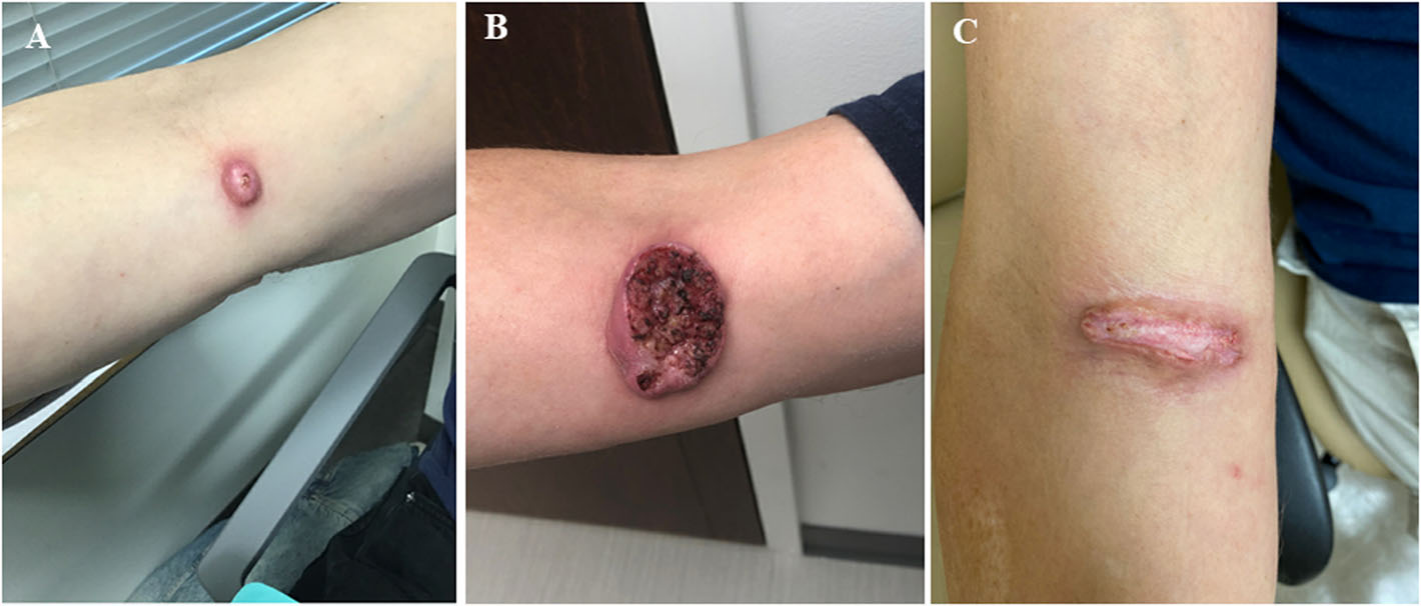
Skin lesions of cutaneous squamous cell carcinoma showing **a** a new dome-shaped nodule in the antecubital fossa with ulcerated center 3 months after disconsolation of pembrolizumab. **b** rapid progression of poorly demarcated ulceration. **c** scar formation and skin regeneration with granulation tissue at the base replacing the previously seen ulcerated lesion after restarting pembrolizumab

Due to the locally advanced nature of the tumor and (MSI-H), the patient was started on pembrolizumab 200 mg intravenously every three weeks and this was continued for 2 years. The patient achieved complete clinical and radiographic response after 4 cycles of therapy and maintained that response throughout his course of treatment (Fig. 2). Three months after discontinuation of pembrolizumab, the patient developed a small nodule in the right antecubital fossa (Fig. 3) and biopsy showed a well differentiated SCC of the skin with PD-L1 expression < 1% (Fig. 1). Given the unusual recurrence of cSCC in a new location and the presence of MLH1 and MSH6 mutations in the tumor with an unclear family history of cancer, germline genetic testing for hereditary cancers was performed after discussion with the patient. This revealed a germline mutation in MLH1 (EX4_8del).

Staging imaging with CT showed no sites of metastatic disease, and the right antecubital lesion was deemed recurrent cSCC. During workup, the lesion progressed very rapidly within 1 month (Fig. 3). We restarted pembrolizumab urgently with rapid observed response after 2 cycles of therapy (Fig. 3). Currently, the patient has been treated for 5 months and maintains his clinical response. The patient was referred for genetic counseling and further evaluation including appropriate cancer screening.

## Discussion and conclusion

Lynch syndrome (LS) is a common inherited disorder involving germline mutation in DNA mismatch repair (MMR) genes [1]. Although the main malignant phenotypic manifestation of LS is colorectal cancer, patients are at increased risk of other visceral tumors such as gastrointestinal, gynecological and genitourinary malignancies [2]. Skin tumors in the context of LS are rare with the most recognized variant being Muir-Torre and Turcot syndromes which can be associated with skin tumors including sebaceous gland tumors and keratoacanthoma [3]. Of interest, the incidence of cutaneous squamous cell carcinoma (cSCC) is very rare and has not been described in detail in the literature. Our report demonstrates the potential risk for developing cSCC in patients with LS and provides an insight into the clinical course of the disease in one patient. In a large prospective cohort of 1942 patients with germline mutations and documented LS, there were only 12 cases with skin cancer including epithelial and sebaceous gland invasive cancer [4]. Our reports highlight a case of a young patient who did not have a history of LS and presented with cSCC in a non- sun exposed area (groin). He did not have risk factors such as immunosuppression or prolonged sun exposure. The patient had a recurrence of his disease after surgical resection with high MSI detected on NGS of tumor tissue, which prompted treatment with pembrolizumab [5]. However, there was a rapid recurrence of cSCC in a different location (arm), which was observed after discontinuation of pembrolizumab. The presence of mutations in MSH6 and MLH1 with high tumor mutational burden prompted further review of patient’s family history and germline mutation testing, which revealed MLH1 germline mutation consistent with LS. Interestingly, we identified two different mutations by NGS on tumor tissue (MLH1 and MSH6), and later the patient was found to have MLH1 germline mutation. We believe that MSH6 mutation which was detected on tissue specimen was a somatic mutation, as it has been described to be present sporadically or in association with different lynch germline mutations [6].

Our case emphasizes the importance of considering hereditary cancer syndromes such as LS in young patients who present with cSCC, and do not display risk factors such as immunosuppression or substantial sun and/or radiation exposure. Some reports described patients with LS with MLH1 and MSH2 germline mutations, who developed cSCC and are summarized in Table 1 [2, 7, 8]. Moreover, there has been reports of patients with cSCC who had MLH1 mutation in their tumor on NGS but there was no consideration of genetic counseling or germline testing in those cases [9]. Another report described a patient with cSCC who had mutations in MSH2, MLH1 and PMS2 with history of LS but with a negative germline mutation testing [10]. Our report, combined with previous observations, suggest the importance of careful examination of patient’s family history and high index of suspicion of hereditary cancer syndromes when patients present with cSCC without known risk factors, especially in young patients as this could be the initial presentation of hereditary diseases such as LS. The consideration of germline mutation testing is reasonable in such cases after discussion with patients about risks and benefits of germline testing and proper genetic counseling. Similarly, the occurrence of cSCC in non- sun exposed skin areas and the recurrent pattern should prompt further consideration of hereditary syndromes including LS. Pembrolizumab, an immune checkpoint inhibitor, has shown efficacy in tumors harboring high MSI, such as the case of our patient, and was also approved recently for the treatment of cSCC [11, 12]. The trial protocols used pembrolizumab until disease progression or high-grade adverse event. In our patient, we observed a rapid response of cSCC in the groin region to pembrolizumab, however, there was a rapid progression of cSCC in a different skin site within 3 months after stopping pembrolizumab. Moreover, restarting the medication resulted in a rapid and sustained response which favors a strategy of continuous administration in such patients.

**Table 1:** Cases describing Lynch syndrome patients with documented germline mutation associated with cutaneous squamous cell carcinoma

Author, Year	Age	Gender	Germline Mutation	Other Malignancies	Treatment of cSCC	Outcome of cSCC
Sorscher S, 2015 [7]	54	Male	MLH1 (1772del4)	Colon Cancer	Resection	N/A
Adan F et al. 2019 [8]	33	Female	MSH2 (c.551del p.Phe184Serfs*30)	None (Hyperplastic colon polyp)	Resection	N/A
Latham A et al. 2019 [2]	69	N/A	MSH2 (c.1046C > G; p.Pro349Arg)	Colon, Prostate, Urothelial	N/A	N/A
Latham A et al. 2019 [2]	55	N/A	MSH2 (c.1906G > C; p.Ala63Pro)	Colon, Urothelial	N/A	N/A
Latham A et al. 2019 [2]	62	N/A	MSH2 (c.1216C > T; p.Arg406*)	Colon, Prostate	N/A	N/A

*cSCC* Cutaneous squamous cell carcinoma, *N/A* not available

In conclusion, we report a young 49-year-old patient who presented with cutaneous squamous cell carcinoma harboring germline mutation in MLH1 which was compatible with Lynch syndrome. This report suggests the following: 1) cSCC, although rare, can be the initial presenting manifestation of LS; 2) LS should be considered in patients who present with unexplained cSCC with a positive family history for caner and without known risk factors for skin cancer, especially in younger patients; 3) discontinuation of pembrolizumab in patients with LS and cSCC could be associated with a rapid recurrence of cSCC.

## Data Availability

Not applicable.

## Abbreviations

LS: Lynch syndrome
PD-1: Programmed death-1
PD-L1: Programmed death- ligand 1
cSCC: Cutaneous squamous cell carcinoma
MMR: Mismatch repair
CT: Computed tomography
